# Ovine and murine T cell epitopes from the non-structural protein 1 (NS1) of bluetongue virus serotype 8 (BTV-8) are shared among viral serotypes

**DOI:** 10.1186/1297-9716-45-30

**Published:** 2014-03-12

**Authors:** José M Rojas, Lourdes Peña, Verónica Martín, Noemí Sevilla

**Affiliations:** 1 Centro de Investigación en Sanidad Animal (CISA-INIA), Instituto Nacional de Investigación Agraria y Alimentaria, Valdeolmos, Madrid, Spain; 2 Present address: Centro Nacional de Biotecnología, CSIC, Campus de Cantoblanco, Madrid, Spain

## Abstract

Bluetongue virus (BTV) is a non-enveloped dsRNA virus that causes a haemorrhagic disease mainly in sheep. It is an economically important *Orbivirus* of the *Reoviridae* family. In order to estimate the importance of T cell responses during BTV infection, it is essential to identify the epitopes targeted by the immune system. In the present work, we selected potential T cell epitopes (3 MHC-class II-binding and 8 MHC-class I binding peptides) for the C57BL/6 mouse strain from the BTV-8 non-structural protein NS1, using H2^b^-binding predictive algorithms. Peptide binding assays confirmed all MHC-class I predicted peptides bound MHC-class I molecules. The immunogenicity of these 11 predicted peptides was then determined using splenocytes from BTV-8-inoculated C57BL/6 mice. Four MHC-class I binding peptides elicited specific IFN-γ production and generated cytotoxic T lymphocytes (CTL) in BTV-8 infected mice. CTL specific for 2 of these peptides were also able to recognise target cells infected with different BTV serotypes. Similarly, using a combination of IFN-γ ELISPOT, intracellular cytokine staining and proliferation assays, two MHC-class II peptides were identified as CD4^+^ T cell epitopes in BTV-8 infected mice. Importantly, two peptides were also consistently immunogenic in sheep infected with BTV-8 using IFN-γ ELISPOT assays. Both of these peptides stimulated CD4^+^ T cells that cross-reacted with other BTV serotypes. The characterisation of these T cell epitopes can help develop vaccines protecting against a broad spectrum of BTV serotypes and differentiate infected from vaccinated animals.

## Introduction

Bluetongue virus (BTV) is the prototype member of the *Orbivirus* genus of the Reoviridae family, transmitted to the vertebrate host by biting midges [1]. The genome consists of ten double-stranded RNA segments, encoding 7 structural- and 4 non-structural- proteins [2,3]. The outer capsid layer includes VP2 and VP5 [4,5] responsible for eliciting serotype-specific neutralising antibodies [6,7]. The non-structural (NS) proteins are involved in the control of BTV replication, maturation and export from the cell [8,9].

A long-lasting immunity is developed in animals that recover from bluetongue where both neutralising antibodies [10] and cytotoxic T lymphocytes (CTL) [11,12] are involved in this protective immunity. However, the variability of the outer capsid of this virus represents one of the major challenges for the development of a vaccine capable of protecting animals against multiple serotypes. On the other hand, cellular immunity plays a key role in BTV immunity as adoptive transfer of lymphocytes could partially protect monozygotic sheep from subsequent BTV challenge [13] and protection can exist in the absence of neutralising antibodies [14,15]. Importantly, the determinants for cellular immunity are more likely to be shared among serotypes. Indeed, BTV vaccination and infection in sheep induces CTLs cross-reactive to multiple serotypes [11,16-18]. Based on this observation, vaccination designed to elicit T cell responses can potentially protect animals against several BTV serotypes.

Analysis of CTL responses to BTV in experimentally infected sheep showed that virtually all animals recognise epitopes within the non-structural protein 1 (NS1) [11]. Thus, we have investigated T cell epitopes from the NS1 protein capable of cross-reacting with multiple BTV serotypes both in sheep and mouse, as murine models of BTV infection represent a valuable tool for designing novel vaccination strategies [19,20]. In the present report we identify novel CD4^+^ and CD8^+^ T cell epitopes in mouse model from the NS1 protein of BTV-8, as well as two immunoreactive CD4 epitopes in BTV-8 infected sheep capable of cross-reacting with other serotypes. This work underlines the potential of stimulating anti-BTV T cells in order to develop more effective vaccinations.

## Material and methods

### Cell lines, virus stock preparation and inactivation

BTV stocks and virus titres were prepared as described previously [21]. Briefly, Baby Hamster Kidney (BHK) cells were infected with BTV at multiplicity of infection of 1 and culture supernatants were collected after 48 h. After 3 cycles of freeze/thaw and a 2-min sonication step, the supernatants were clarified by centrifugation and stored at -80 °C until use. Virus titres were determined using a standard plaque titration assay using the Vero cell line. Inactivated virus (BEI-BTV) were obtained by incubating viral stocks (1 × 10^6^ plaque forming unit (pfu)/mL) for 48 h at 37 °C with 3 mM of freshly prepared binary ethyleneimine (BEI) and neutralised with 0.02 M sodium thiosulphate at the end of the incubation time.

### Infections and animals

Female (7–12 week-old) C57BL/6 mice (Harlan Interfauna Ibérica, Barcelona, Spain) were inoculated subcutaneously with 100 pfu of BTV-8 (Belgium/06) three times at 10 days intervals and sacrificed three days after the last inoculation. Three month-old female sheep (Mallorquina breed) (*n* = 8) were inoculated three times at 28-day intervals with 1 × 10^5^ pfu BTV-8. Venous blood was collected 14 days after the last inoculation and peripheral blood leukocytes (PBL) were prepared as described below. All the procedures herein described were carried out under European Community guidelines and approved by the local ethical review committee.

### Peptides and peptide binding assays

Peptide binding assays to D^b^ and K^b^ molecules were performed as previously described [21]. Putative binding peptides for D^b^/K^b^/I-A^b^ molecules from the NS1 protein of BTV-8 were selected using a combination of predictive algorithms available on the web [22-25] (Table 1). Peptides were purchased from Altabiosciences (Birmingham, UK) or Thermo Fisher Scientific (Ulm, Germany). The gp(33–41) peptide (KAVYNFATC) from lymphocytic choriomeningitis virus (LCMV) known to bind D^b^ and K^b^ molecules was used as irrelevant peptide where mentioned. Assays were performed at least 3 times and binding affinity was ranked according to peptide gp(33–41). All peptides were dissolved in DMSO and controls with equivalent amounts of DMSO were included in all experiments.

**Table 1:** **Prediction of H-2**^**b **^**binding peptides from NS1 and binding assays**

				**Predictive algorithm scores**			
**Position**	**Name**	**Sequence**	**Predicted allele binding**	**SYFPEITHI score (D**^**b**^**/K**^**b**^**)**	**NetMHC predicted affinity nM (D**^**b**^**/K**^**b**^**)**	**BIMAS proPred-I score (D**^**b**^**/K**^**b**^**)**	Binding assay* (D^**b**^**/K**^**b**^**)**
14	NS1(14)	**YANATRTFL**	D^b^	16	341	17.892	+
125	NS1(125)	**SALVNSERV**	D^b^	28	10	138.526	+++
152	NS1(152)	**GQIVNPTFI**	D^b^	28	5	1502.928	+++
222	NS1(222)	**IQLINFLRM**	D^b^/K^b^	22/17	239/201	226.359/11.000	++/++
403	NS1(403)	**NCYTGAEAL**	D^b^/K^b^	14/11	21953/18590	3.982/6.000	+/-
141	NS1(141)	**AMPYIYVPI**	K^b^	22	137	19.800	+
166	NS1(166) Cl I	**IAYYFYNPD**	K^b^	19	30	1.320	+++
235	NS1(235)	**KHFNRYASM**	K^b^	17	22	40.000	+++
166	NS1(166) Cl II	**IAYYFYNPDAADDWI**	I-A^b^	NA	194.8	NA	NA
402	NS1(402)	**TNCYTGAEALITTAI**	I-A^b^	NA	87.3	NA	NA
522	NS1(522)	**KVHFAGFAAPACESG**	I-A^b^	NA	59.1	NA	NA

NA: Not available.

*Peptides were ranked relative to the binding of the control peptide gp33-41 from LCMV to Db and Kb molecules on RMA-s cells (fluorescence ratio between NS1 peptide and gp33-41 peptide) (Rojas et al. [21]). **+++**: strong binder where ratio > 0.9; **++**: moderate binder where ratio > 0.7 and < 0.9; **+**: weak binder where ratio > 0.5 and < 0.7; and **-**: no binder where ratio < 0.5.

### Splenocytes and lymph node cells preparation

Spleen and mesenteric lymph nodes were collected from inoculated mice three days after the last inoculation. Single cell suspensions were prepared by mechanical disruption of the organs through a cell strainer. After lysing the erythrocytes, splenocytes or lymph node cells from each mouse were tested against the appropriate stimuli individually at least in triplicate in T cell medium (RPMI supplemented with 10%FCS (Lonza Biowhittaker, NJ, USA), 4 mM L-glutamine, 10 mM HEPES, 1% 100X non-essential amino-acids, 1 mM sodium pyruvate, 100U/mL penicillin/100 μg/mL streptomycin and 50nM β-mercaptoethanol (all from Gibco, Invitrogen)).

### PBL preparation and in vitro stimulation

PBL were prepared by standard centrifugation method. Briefly, venous blood collected in EDTA (6 mM final concentration) was diluted 1:1 in PBS + 0.03% (w/v) EDTA (pH 7.4) and overlayed over a Ficoll cushion (GE Healthcare Europe GmbH, Barcelona, Spain). Blood was centrifuged at 800 × *g* for 30 min at room temperature without brake, and the PBL present at the interface were transferred to a fresh tube and washed with PBS + 0.03% (w/v) EDTA. Contaminant erythrocytes were lysed and after two further washes, cells were cryopreserved in 90% FCS + 10% DMSO until use. Sheep PBL were thawed by slowly diluting the cryovial content into PBL medium (RPMI + 17% AIM-V medium + 5% FCS + 4 mM L-Glutamine + 10 mM HEPES + 1% 100X non-essential amino-acids + 1 mM sodium pyruvate + 100U/mL penicillin/100 μg/mL streptomycin + 50 nM β-mercaptoethanol (all from Gibco, Invitrogen, CA, USA)). Sheep PBL were washed three times and rested for 1–2 h at room temperature in PBL medium before use. In some experiments, 5 × 10^6^ sheep PBL per well were restimulated with 10 μg/mL of NS1 peptide in 24 well plates for 7 days prior to flow cytometry analysis for intracellular IFN-γ.

### ELISPOT assays

Murine IFN-γ ELISPOT assays were performed according to the manufacturer protocol (Diaclone, France). Briefly, 2 × 10^5^ splenocytes per well were plated in the presence of 10 μg/mL peptide (final concentration) or chemically-inactivated BTV (BEI-BTV). Ovine IFN-γ ELISPOT assays were performed using MSIPS4510 plate (Millipore). Membranes were incubated at 4 °C with 5 μg/mL anti-ovine IFN-γ antibody (MT17.1, Mabtech, Sweden). Sheep PBL were plated at a density of 2–3 × 10^5^ cells per well and incubated with peptide (10 μg/mL), BEI-BTV, PBL medium as negative control or concanavalin-A (0.5 μg/mL) as positive control. Membranes were incubated with biotin-labelled anti-ovine IFN-γ antibody (MT307-biotin, Mabtech, Sweden) and developed with streptavidin conjugated to alkaline phosphatase (ExtrAvidin-AP, Sigma, USA). Membranes were revealed using SigmaFAST BCIP/NBT (Sigma).

### Proliferation assays and intracellular cytokine staining

Splenocytes or sheep PBL (2–3 × 10^5^ per well) were cultured in the presence of BEI-BTV or NS1 peptides (10 μg/mL). For proliferation assay, cells were cultured for 72 h before ^3^H-Thymidne was added to each well and incubated overnight. Data are presented as stimulation index (ratio of incorporated ^3^H-thymidine in test to control cultures) or as average cpm. For intracellular cytokine staining, splenocytes were stained with anti-mouse CD4-FITC and anti-mouse CD8α-PcP antibodies (both from BD Pharmingen, CA, USA) whereas sheep PBL were stained with anti-ovine CD4-FITC and anti-ovine CD8-PE antibodies (both from Serotec). After permeabilisation, splenocytes were stained with anti-mouse IFN-γ-PE and anti-mouse TNF-α-APC (both from BD pharmingen), whereas sheep PBL were stained with anti-ovine IFN-γ-A647 (Serotec, UK).

### Cytotoxicity assays

CTL assay was quantitated by a standard ^51^Cr-release assay [21,26]. Target cells (T) (RMA-S or MC57 cells) cells were pulsed for 1 h in serum-free medium at 37 °C, 5% CO_2_ with 10 μg/mL of peptide (NS1 or irrelevant gp33-41 peptide), BEI-BTV (equivalent to 5 × 10_5_ PFU/mL prior to inactivation) or BHK-mock lysate as negative control. Target cells (T) were labelled with 50 μCi of Na_2_^51^CrO_4_ (Hartmann Analytic, Germany). Effector (E) (splenocytes) and target cells were cultured in triplicates at different ratios Culture supernatants were counted using a 1450 MicroBeta Trilux counter (Perkin-Elmer, Ohio, USA). The specific percentage lysis was calculated using the following formulae:

$$\begin{array}{ll} \text{Percentage}\;\text{cytotoxicty}= & \frac{\left(\text{Experimental}\;\text{release}‒\text{Spontaneous}\;\text{release}\right)}{\left(\text{Maximum}\;\text{release}‒\text{Spontaneous}\;\text{release}\right)}\\ & \;\times 100 \end{array}$$

### Statistical analysis

Unpaired two-tailed Student’s *t*-tests were used to observe difference within the same animal, whereas non-parametric two-tailed Mann Whitney rank *U* tests were used to compare group of values for several animals. Data handling analyses was performed using Prism 5.0 (GraphPad Software Inc. San Diego, CA, USA).

## Results

### Infection with BTV-8 induces T cell responses to other BTV serotypes in mice and sheep

**Figure 1:**
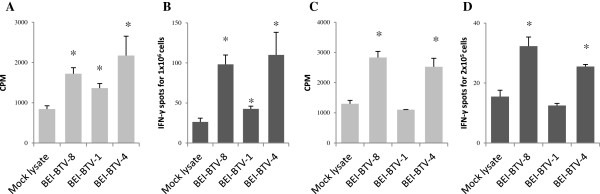
**T cells from BTV-8 inoculated animals recognise antigenic determinants shared by BTV serotypes.** C57BL/6 mice inoculated were sacrificed and splenocytes were cultured in the presence of inactivated BTV from different serotypes. **(A)** Proliferative responses to BTV were detected not only to the serotype used for vaccination (BTV-8) but also to serotype 1 and 4. **(B)** Similarly, splenocytes produced IFN-γ specifically when in presence of BTV serotype 1, 4 and 8. Data presented are representative of 5 mice inoculated with BTV-8. **(C)** PBLs from BTV-8 infected sheep were cultured for 5 days in the presence of inactivated BTV from different serotypes and tritiated-thymidine was added in the last 16 h of culture. Proliferative response to BTV-4 and BTV-8 but not BTV-1 was detected in the cultures. Data presented are representative of 6 sheep. **(D)** PBLs from BTV-8 infected sheep were cultured with inactivated BTV from different serotypes for 48 h and IFN-γ was measured by ELISPOT. IFN-γ was produced to BTV-8 and BTV-4 but not BTV-1. Data presented are representative of 3 sheep. Proliferation is presented as the average cpm of ^3^H-thymidine incorporated in triplicate cultures and IFN-γ ELISPOT assays as average number of spots in triplicate cultures. *denotes *p* < 0.05 using an unpaired Student *t* test (control vs BTV).

Splenocytes from BTV-8 inoculated mice were cultured in the presence of inactivated virus. Proliferation (Figure 1A) and IFN-γ (Figure 1B) were not only detected to inactivated BTV-8 but also to inactivated BTV-4 in all 5 mice tested and to inactivated BTV-1 in 4 out of 5 mice. In sheep experimentally infected with BTV-8, we detected proliferative responses and IFN-γ production not only to BEI-inactivated BTV-8 but also to BTV-4. However no proliferative response or specific IFN-γ production to BTV-1 was detected (Figures 1C and 1D). It remains unclear whether this difference in response is due to a different antigen repertoire presented to the host, or to a mechanism of immune evasion specific to BTV-1. Taken together, these results confirm that T cells from infected animals can cross-react with several serotypes.

### Prediction of peptide binding from NS1 protein to H-2^b^ haplotype and binding assays

Using a combination of three epitope prediction algorithms available on the web (SYFPEITHI, Pro-Pred-I, NetMHC I and II) [22-25], 11 peptides from NS1 protein from BTV-8 were selected and synthesised (Table 1). The ability of these synthesised peptides to bind H-2 D^b^ and K^b^ molecules was assessed using a binding assay on RMA-S cells. All 8 peptides predicted to bind murine MHC class I molecules showed binding affinity for either D^b^ or K^b^ molecules. NS1 peptide binding was ranked according to the binding to RMA-S cells of the control peptide gp33-41. Four NS1 peptides displayed a strong affinity for either D^b^ or K^b^ molecules, and conversely, 3 NS1 peptides only had low affinity for their MHC molecules (Table 1). Peptide NS1(222) displayed, as predicted, moderate binding to both D^b^ and K^b^ molecules. Overall, the NetMHC algorithm appears to predict more accurately the peptide binding to D^b^ and K^b^ molecules, although NS1(403) peptide which was not predicted to bind using this algorithm had weak affinity for D^b^ in RMA-S cell binding assays. Thus, we can conclude that the combination of several predictive algorithms proved useful for the detection of putative T cell epitopes.

### Response to D^b^ and K^b^ binding NS1 peptides

**Figure 2:**
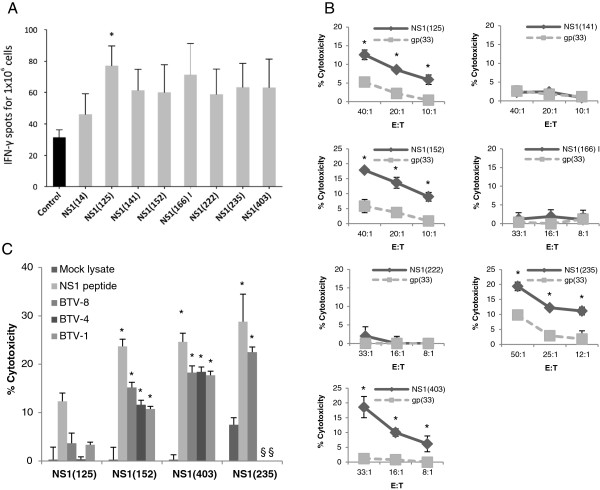
**Response to D**^**b **^**and K**^**b **^**binding NS1 peptides. (A)** Splenocytes were cultured with NS1 peptides for 48 h and IFN-γ production was measured using ELISPOT assays. Peptides NS1(141/152/166/222/403) showed specific IFN-γ in only 3 or 4 mice out of the 6 mice used in these experiments. Peptide NS1(14) was only positive in one mouse, whereas peptide NS1(125) was positive for IFN-γ production in 5 out of 6 mice. (*p < 0.05 Mann–Whitney test NS1(125) vs control). Data are presented as the mean number ± SD of IFN-γ-producing cells in response to stimuli in 6 mice. **(B)** CTL lines were generated by in vitro NS1 peptide stimulation (effector: E) and tested against RMA-S cells (target: T) pulsed with the NS1 peptide used for in vitro stimulation or with the control peptide gp(33). Peptides NS1(125/152/235/403) generated specific CTL responses, whereas no CTLs were generated using NS1(141/166(I)/222) peptides. Data presented are representative of at least 4 mice for each peptide (*p < 0.05 unpaired Student *t* test NS1 peptide vs control gp(33) peptide). **(C)** CTL lines specific for NS1(125/152/235/or 403) peptide were also tested against MC57 cells as target (E:T = 50:1) pulsed with relevant NS1 peptide as positive control, mock BHK lysate as negative control, or BEI-BTV-1, 4 or 8 to assess epitope processing. CTLs specific for NS1(152) and NS1(403), but not NS1(125) peptide, were capable of recognising target cells pulsed with BTV from different serotypes (*p* < 0.05 unpaired Student *t* test: mock lysate vs NS1 peptide, BTV-1, 4 or 8). CTLs specific for NS1(235) peptide were able to recognise target cells pulsed with BTV-8. However due to the limited number of CTL obtained in these experiments, we were not able to confirm the expression of this epitope in BTV-1 or 4. All CTL data presented are representative of at least 4 mice for each peptide. §: not done.

Splenocytes from BTV-8 inoculated mice were cultured for 48 h in the presence of NS1 peptides and the specific IFN-γ production was assessed using ELISPOT assays (Figure 2A). Only peptide NS1(125) showed a significant IFN-γ production in 5 out of 6 mice tested, which was significant using a Mann Whitney rank test comparing the NS1(125) peptide group to the control group. Peptides NS1(141), NS1(152), NS1(166) I, NS1(222), NS1(235) and NS1(403) showed significant IFN-γ production in 3 or 4 mice out of 6 (by student *t* test), whereas peptide NS1(14) induced specific IFN-γ production in only 1 mouse out of 6 and thereby was not investigated further. We then proceeded to generate CTL lines specific for these NS1 peptides from splenocytes of inoculated mice. After 5–6 days of in vitro stimulation with NS1 peptide, splenocyte cultures were tested for cytotoxicity by ^51^chromium release assay against RMA-S cells either pulsed with the NS1 peptide of interest or gp33-41 peptide as control (Figure 2B). Peptides NS1(125), NS1(152), NS1(235) and NS1(403) generated CTLs in BTV-8 inoculated mice, whereas no evidence of specific CTL activity was detected to NS1(141), NS1(166) I, or NS1(222) peptides. Taken together, these data suggest that NS1(125), NS1(152), NS1(235) and NS1(403) peptide are CTL epitopes from NS1 in the context of H-2^b^.

To analyse whether these 4 peptides were naturally processed and presented by target cells cultured with BTV (Figure 2C). MC57 cells were incubated with BEI-BTV either from serotype 1, 4 or 8 and used as target cell for CTL raised against NS1(125), NS1(152), NS1(235) or NS1(403) peptide. MC57 cells were pulsed with the relevant NS1 peptide as positive control, or with mock BHK lysate as negative control. CTLs specific for NS1(152), NS1(235) or NS1(403) peptide were able to lyse target cells cultured with BTV-8. However, CTLs specific for NS1(125) peptide failed to recognise target cells cultured in the presence of BTV. Importantly, CTLs specific for NS1(152) or NS1(403) peptide were capable of lysing target cells pulsed either with BTV-1 or BTV-4, demonstrating that these CTLs can recognise cells infected with different BTV serotypes. Therefore, NS1(152) and NS1(403) peptides are naturally processed by other BTV serotypes and are capable of inducing cross-reactive CTL responses.

### Response to I-A^b^ binding peptides

**Figure 3:**
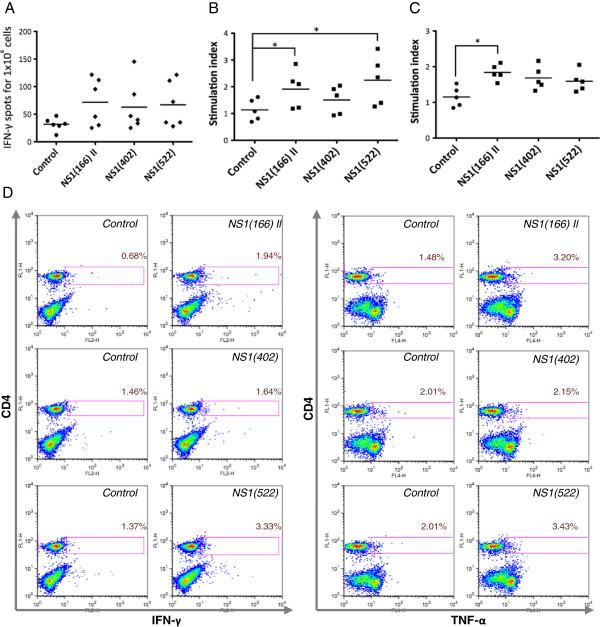
**NS1(166) II and NS1(522) peptides are CD4 epitopes in C57BL/6 mice. (A)** No consistent significant production of IFN-γ was detected by ELISPOT to any peptide using a Mann–Whitney test. However, in individual mice significant IFN-γ production (using an unpaired Student’s *t* test) was detected in 3 out of 6 BTV-8-inoculated mice to peptides NS1(166) II and NS1(522) and in 2 out of 6 mice to peptide NS1(402). **(B)** Lymph node cells from BTV-8 inoculated mice proliferated significantly to both NS1(166) II and NS1(522) peptides. * *p* < 0.05 Mann–Whitney test on NS1 peptide vs control. **(C)** Splenocytes from BTV-8-inoculated mice consistently proliferated to the NS1(166) II peptide, whereas NS1(402) and NS1(522) peptides showed no specific proliferation. * *p* < 0.05 Mann–Whitney test on NS1 peptide vs control. **(D)** Using intracellular cytokine staining, we observed that IFN-γ and TNF-α were produced by CD4+ T cells when cultured in the presence of NS1(166) II and NS1(522) peptides, but not with NS1(402) peptide. Dot-plots are representative of at least 3 different mice. Percentages indicate the number of cytokine producing cells in the CD4+ population.

Production of IFN-γ to putative I-A^b^ binding peptides from NS1 was measured by ELISPOT assays on splenocytes from BTV-8 inoculated mice (Figure 3A). No peptide displayed consistent production of IFN-γ in these assays, but peptides NS1(166) II and NS1(522) showed significant cytokine production (by Student’s *t* test) in 3 out of 6 mice tested. Proliferation was measured using ^3^H-thymidine incorporation in lymph node cells (Figure 3B) and splenocytes (Figure 3C) from BTV-8 inoculated C57BL/6 mice cultured for 72 h in the presence of NS1 peptides. Peptide NS1(166) II showed significant proliferative response in splenocytes (Mann Whitney test) whereas peptides NS1(402) and NS1(522) did not displayed significant and consistent proliferative responses in spleen (Figure 3C). In lymph nodes, peptides NS1(166) II and NS1(522) showed significant proliferative response (Figure 3B). Moreover, intracellular staining for IFN-γ and TNF-α demonstrated by flow cytometry that CD4+ T cells responded to NS1(166) II and NS1(522) peptides, but not to NS1(402) peptide (Figure 3D). Taken together, these data indicated that NS1(166) II and NS1(522) peptides are CD4 epitopes from BTV-8 in C57BL/6 mice.

### Response to predicted NS1 peptides in sheep

**Figure 4:**
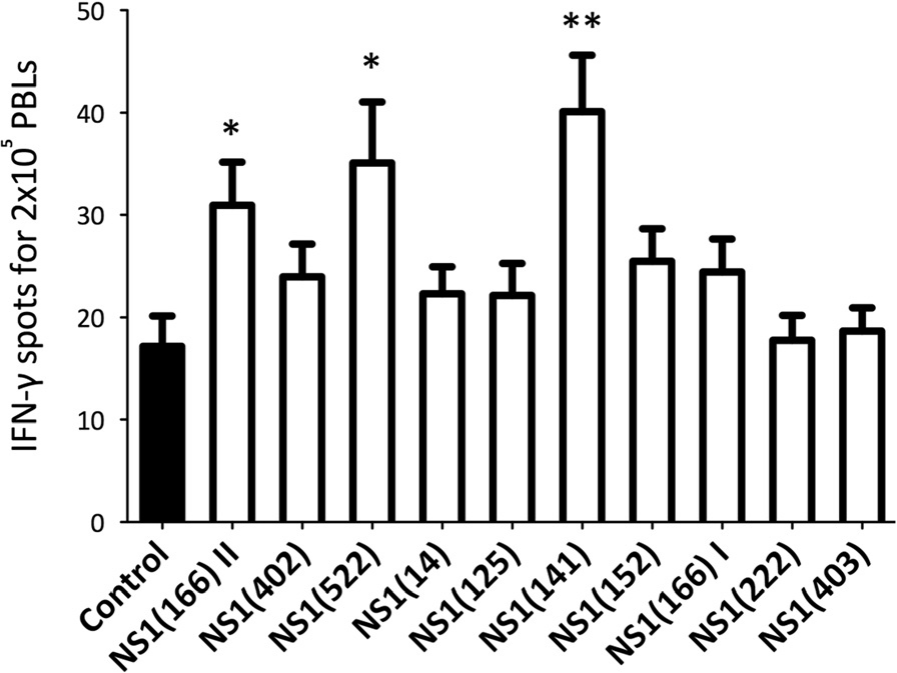
**Sheep PBL responses to NS1 predicted peptides.** PBLs from sheep infected experimentally with BTV-8 were cultured for 48 h with NS1 peptides and specific IFN-γ response was measured using an ELISPOT assay. Peptide NS1(141) and NS1(522) showed specific IFN-γ production in 7 out of 8 sheep. Peptide NS1(166) II showed specific IFN-γ production in 5 out of 8 sheep. Data are presented as the mean number ± SD of IFN-γ-producing cells in response to stimuli in 8 sheep. *p < 0.05 and **p < 0.01 Mann Whitney test NS1 peptide group vs control.

**Figure 5:**
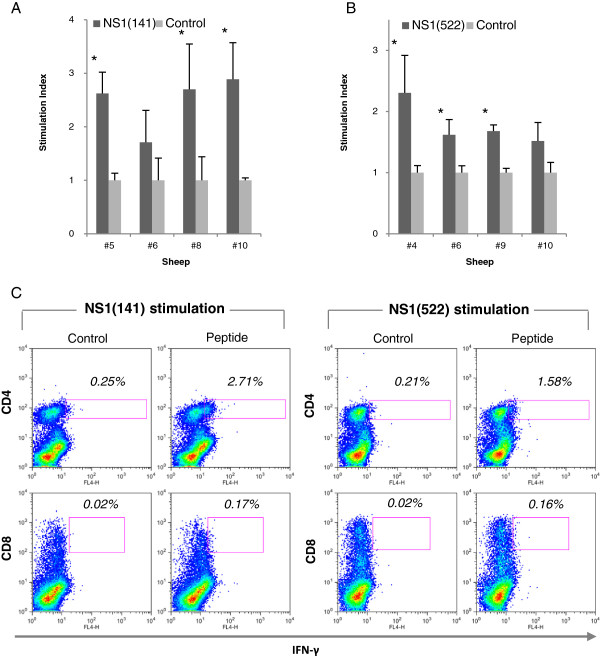
**Peptides NS1(141) and NS1(522) are CD4 epitopes in sheep.** PBLs from sheep infected experimentally with BTV-8 were cultured for 5 days with **(A)** NS1(141) or **(B)** NS1(522) peptides and proliferative responses were measured using ^3^H-thymidine incorporation. Three out of 4 sheep tested had specific proliferative responses to these peptides. Data are presented as stimulation index representing the ratio of cpm between peptide stimulated PBLs vs DMSO control culture PBLs. * *p* < 0.05 Unpaired Student *t* test NS1 peptide vs control. **(C)** To determine whether the proliferative response and IFN-γ production to these NS1 peptide in sheep was mediated by CD4+ or CD8+ T cells, intracellular IFN-γ staining of peptide-stimulated sheep PBL was performed and data were analysed by flow cytometry. The production of IFN-γ to NS1(141) and NS1(522) peptides was detected in CD4+ T cells but not in CD8+ T cells. Data presented are representative of 3 sheep.

To determine whether some degree of overlap in these epitopes is present in BTV-8-infected sheep, we stimulated in vitro PBL from sheep infected experimentally with BTV-8 with NS1 peptides. After 48 h, the specific IFN-γ response was assessed using an ELISPOT assay (Figure 4). Peptides NS1(141) and NS1(522) showed significant IFN-γ production in most infected sheep. Peptide NS1(166) II was also immunogenic but to a lesser extent (in 5 out of 8 sheep tested). Therefore, we further investigate peptides NS1(141) and NS1(522). PBL from infected sheep were cultured in the presence of NS1 peptide, and proliferation was assessed using ^3^H-thymidine incorporation at day 5 (Figures 5A and 5B). Peptide NS1(141) and NS1(522) induced proliferative response in 3 out of 4 tested sheep. These data confirmed that NS1(141) and NS1(522) peptides are immunogenic in sheep. To determine whether NS1(141) and NS1(522) peptides elicit CD4^+^ or CD8^+^ T cell responses, sheep PBL were stimulated with peptide and intracellular IFN-γ was measured by flow cytometry (Figure 5C). For both peptides, IFN-γ was detected in CD4^+^ T cells but not in CD8^+^ T cells. Thus, these data indicate that NS1(141) and NS1(522) peptides are CD4 epitopes in sheep.

**Figure 6:**
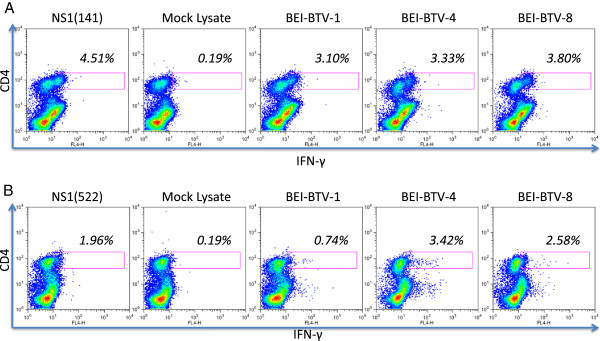
**Peptide NS1(141) and NS1(522) are presented by several BTV serotypes.** PBLs from infected sheep were stimulated either with **(A)** NS1(141) or **(B)** NS1(522) peptide. After 1-week expansion, IFN-γ production was assessed against several BTV serotypes. CD4+ T cells specific for NS1(141) or NS1(522) were able to produce IFN-γ not only in the presence of BTV-8 (used for infection) but also for BTV-1 and -4.

To establish whether these CD4^+^ T cells specific for NS1(141) or NS1(522) peptide can cross-react with several BTV serotypes, sheep PBL stimulated with these peptides were cultured in the presence of BEI-BTV from different serotypes (Figure 6). In some cases, BEI-BTV stimulation resulted in IFN-γ production in the CD4 negative compartment to all 3 serotype tested, probably as a result of antigen recognition by BTV-specific CD8 + T cells still present in the PBL cultures. This suggests that CD8 epitopes can also be shared among serotypes in sheep. Cells specific for both peptides were capable of producing IFN-γ not only to the serotype used for inoculation (BTV-8) but also to BTV-1 and BTV-4. Therefore, these data indicate that NS1(141) and NS1(522) are CD4 epitopes shared among several BTV serotypes.

## Discussion

In the present work, we described CTL and T helper epitopes from the NS1 protein of BTV-8 in mice and sheep. Three CTL and 2 helper epitopes were described in C57BL/6 mice, and 2 helper epitopes were described in sheep. These T cells are naturally activated during BTV-8 infection and are likely to contribute to the elimination of the virus. As previously described [18], we confirmed that T cells can cross-react with other BTV serotypes. Interestingly, IFN-γ production to BTV-1 was always lower when compared to BTV-8 or BTV-4, indicating either that this strain can interfere with IFN-γ production or that BTV-1 may not share some antigenic determinants responsible for the IFN-γ production observed with its other two serotypes. It remains unclear whether the different response to BTV-1 observed in these experiments was due to a different antigen repertoire presented to the host, or to a mechanism of immune evasion specific to BTV-1.

Murine models for BTV infection are well established [19,27] and represent a very useful tool to design more rational and cost-effective vaccination strategies for the disease. The description of T cells determinants in mice will therefore allow for a better monitoring of effective vaccination to the virus in these experimental models. In turn, this is likely to further improve the design of novel vaccination approaches in the natural host. Predictive algorithms for MHC binding peptides have proved useful in identifying T cell epitopes for virus or tumour antigens [28]. In the present work, we have used a combination of algorithms available on the web to predict H-2^b^ binding peptides from NS1. The binding to MHC class I molecules of these predicted peptides was verified using binding assay with RMA-S cells [21,29]. The NetMHC algorithm appears to predict more accurately the binding to D^b^ and K^b^ molecules than the other 2 algorithms. However, NS1(403) peptide which was a predicted binder using SYFPEITHI and Pro-Pred-I algorithms but not by the NetMHC algorithm showed weak affinity for D^b^ in RMA-S cell binding assays. Importantly, in spite of its weak binding NS1(403) peptide was a CTL epitope shared among several BTV serotypes. On the other hand, NS1(125) peptide which showed strong binding to D^b^ could elicit IFN-γ production but displayed only a weak CTL activity in inoculated mice, with no evidence of natural processing in BTV-infected cells. Taken together these data confirm that MHC binding affinity alone is not always a good predictor of CTL activity, and that immune responses may be directed to low affinity peptides [30].

The immunogenicity of these predicted peptides was assessed in BTV-8 inoculated mice using a combination of IFN-γ ELISPOT assays, intracellular cytokine staining, CTL assays (for MHC class I binding peptides) and proliferation assays (for MHC class II binding peptides). Only NS1(125) peptide displayed consistent IFN-γ production in all inoculated mice. However, this peptide only displayed weak CTL activity and no evidence of natural processing was observed in cell pulsed with BTV. Peptides NS1(152), NS1(235) and NS1(403) induced moderate IFN-γ production, however they displayed consistent CTL activity and were able to lyse target cells pulsed with BTV. Two CD4 epitopes were also identified in C57BL/6 inoculated mice using a combination of proliferation assay and intracellular cytokine staining. These data highlight the importance of using several complementary techniques to identify novel epitopes.

**Figure 7:**
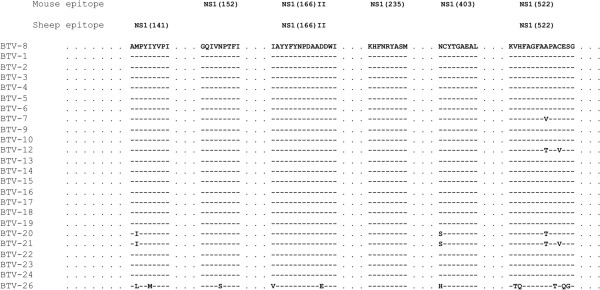
**Murine and ovine T cell epitopes from NS1 have identical sequence in most BTV serotypes.** Sequence identities were searched using BLASTp. Dashes indicate identical amino-acid residues in BTV-8 and the compared sequence. Where residues differed, the single letter code for amino-acid residues is used to indicate the difference.

Importantly, we were able to show that peptides NS1(152) and NS1(403) could recognised cells pulsed with different BTV serotypes demonstrating that these CTL epitopes are presented by other viral serotypes. In the case of CTL raised against NS1(235) peptide, we were not able to use target cells pulsed with other BTV serotypes due to the limited amount of cells obtained in these experiments. Nonetheless, it is very likely that NS1(235) will also be presented by other BTV serotypes as its sequence is identical in BTV-1 and BTV-4. Moreover, the sequences from these NS1 epitopes are shared among a wide variety of BTV serotypes (Figure 7). Thereby, vaccination activating T cells responses to these epitopes is likely to set the basis for a better protection across BTV serotypes.

We also describe two CD4 epitopes from NS1 in sheep. Both of these helper epitopes were not only presented by BTV-8, but also by BTV-1 and BTV-4. Definition of these cross-reactive epitopes is an essential part of the ongoing effort to improve BTV vaccination and monitoring. From a biological point of view, defining these T cell epitopes will help understand the process of infection of BTV, as well as its interaction with the host immune system. The susceptibility that BTV displays to type I interferon as well as the lymphopenia observed in the host after infection, indicate that BTV is immunosuppressive [31]. Animals that recover from the infection develop a strong humoral and cellular immunity to the virus, demonstrating that effective immunity to BTV can be eventually mounted. However during the infection period, animals are immune-compromised and therefore susceptible to opportunistic infections. A better understanding of the T cell response to BTV may be central to comprehend the mechanisms through which this virus is capable of evading the host immune response and often persist for month in the host [32].

The characterisation of more T cell determinants from BTV in breeds used widely in sheep farming is also important for the monitoring of the health status of naïve populations to BTV. In addition, this knowledge may help understand the susceptibility of these breeds to BTV outbreaks. Vaccination designed to activate T cells specific for determinants shared among serotypes is also likely to limit the economical impact that BTV outbreaks can have on naïve populations.

## Competing interests

The authors declare that they have not competing interests.

## Authors’ contributions

JMR, carried out most of the experiments described in the manuscript and wrote the article; LP participated in the animal handling; VM, carried out some of the experiments; NS, conceived the study and contributed in its design and coordination. All authors read and approved the final manuscript.
